# Chemical Profiling of Xueshuan Xinmaining Tablet by HPLC and UPLC-ESI-Q-TOF/MS

**DOI:** 10.1155/2018/2781597

**Published:** 2018-10-21

**Authors:** Ya-Xin Han, Peng-Fei Wang, Meng Zhao, Liang-Mian Chen, Zhi-Min Wang, Xiao-Qian Liu, Hui-Min Gao, Mu-Xin Gong, Hui Li, Ji-Zhong Zhu, Chuan-Gui Liu

**Affiliations:** ^1^School of Traditional Chinese Medicine, Capital Medical University, Beijing 100069, China; ^2^Institute of Chinese Materia Medica, China Academy of Chinese Medical Sciences, National Engineering Laboratory for Quality Control Technology of Chinese Herbal Medicine, Beijing 100700, China; ^3^Tianjin University of Traditional Chinese Medicine, Tianjin 300193, China; ^4^College of Pharmacy, Henan University of Traditional Chinese Medicine, Zhengzhou 450008, China; ^5^Jilin Huakang Stock Ltd., Company of Medicines, Dunhua 133700, China

## Abstract

Xueshuan Xinmaining Tablet (XXT) is a widely used traditional Chinese medicine for the treatment of stroke, chest pain, coronary heart disease, and angina pectoris caused by blood stasis. Having a multiple-component preparation, it is still far from meeting the requirements of modernization and standardization because its detailed chemical basis and action mechanism have not been clarified. In this work, the different batches of XXT samples were analyzed by HPLC and the typical sample was analyzed by UPLC-ESI-Q-TOF/MS to understand its chemical profiling. As a result, 77 chromatographic peaks were detected, among which 63 constituents were identified or tentatively characterized based on the comparison of retention time and UV spectra with authentic compounds as well as by summarized MS fragmentation rules and matching of empirical molecular formula with those of published components. This is the first systematic report on the chemical profiling of the commercial XXT products, which provides the sufficiently chemical evidence for the global quality evaluation of XXT products.

## 1. Introduction

Xueshuan Xinmaining Tablet (XXT) is a widely used traditional Chinese medicine for the treatment of stroke, chest pain, coronary heart disease, and angina pectoris caused by blood stasis [1]. It is prepared from ten raw materials, including Chuanxiong (*Chuanxiong rhizoma*), Huaihua (*Sophorae flos*), Danshen (*Salviae miltiorrhizae radix et rhizoma*), Shuizhi (*Hirudo*), Maodongqing (*Hairy holly root*), Rengong Niuhuang (*Bovis calculus artifactus*), Rengong Shexiang (*Moschus artifactus*), Renshen Jingye Zongzaogan (Total ginsenoside of ginseng stems and leaves), Bingpian (*Borneolum syntheticum*), and Chansu (*Bufonis venenum*) [2]. Nowadays, several qualitative analyses have been reported concerning volatile constituents by GC-MS [3] and chemical fingerprints by HPLC-ELSD [4] and UPLC-PDA [5]. However, having a multiple-component preparation, it is still far from meeting the requirement of modernization and standardization because its complicated ingredients lead to difficulty in clarifying therapeutic material basis, establishing quality control strategy, or selecting chemical markers for pharmacokinetic study.

Ultraperformance liquid chromatography coupled with electrospray ionization tandem quadrupole time-of-flight mass spectrometry (UPLC-ESI-Q-TOF/MS) has become one of the most effective tools for the on-line structural elucidation of multiple components of TCM, owing to its characteristics of accurate mass measurement, high resolution, and excellent sensitivity. MS^E^ (E represents collision energy) technology provides an automated strategy to decrease analysis time and maximize duty cycles by using parallel alternating scans at low collision energy in the collision cell to obtain precursor ion information or at high collision energy to obtain accurate full-scan mass fragment, precursor ion, and neutral loss information. Therefore, both precursor and fragmentation data in exact mass mode were collected in a single run. This method has been proved to provide excellent chromatographic and MS efficiencies in the previous publications [6]. In the present investigation, a combination of HPLC and UPLC-Q-TOF/MS analyses was employed to find out and identify the common chemical profile in various batches of XXT samples. A total of 63 constituents were identified or tentatively characterized based on the comparison with reference substances, on-line UV spectra, and the fragmentation rules.

## 2. Materials and Methods

### 2.1. Materials and Reagents

**Figure 1:**
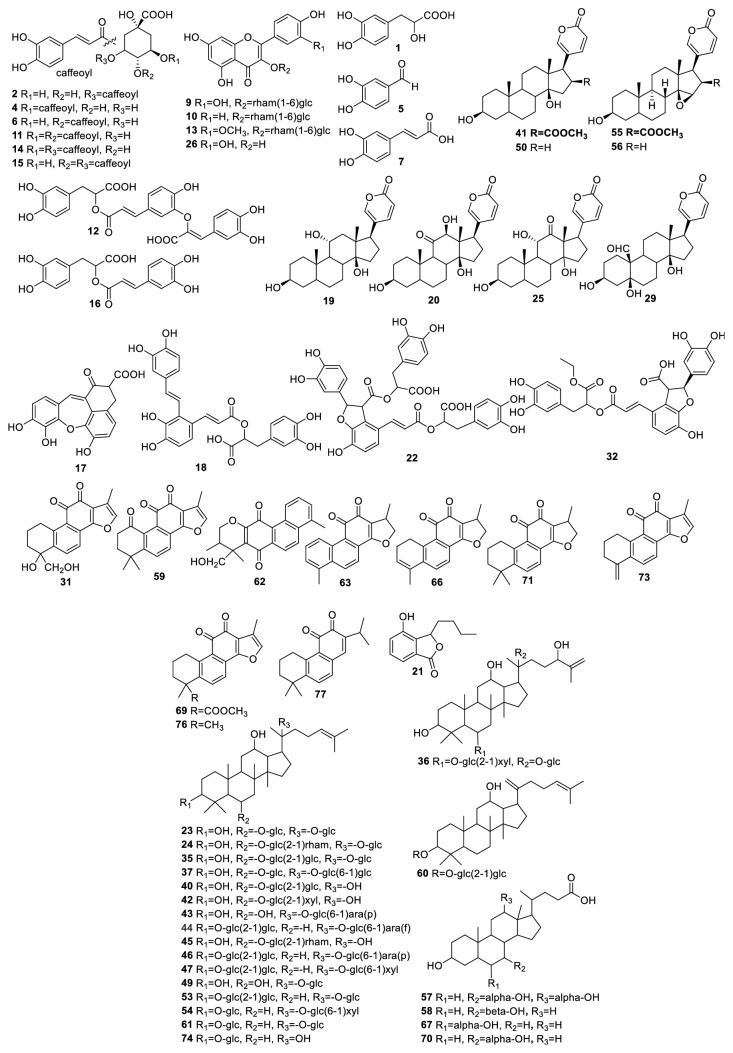
Structures of the identified compounds in XXT sample.

Ten batches of Xueshuan Xinmaining Tablet (Lot. 160904, 160905, 161001-161008) were provided by* Jilin Huakang Stock Ltd., Company of Medicines* (Jilin, China). Reference substances of sodium danshensu (Lot. ZZS17032207), rosmarinic acid (Lot. ZZS17032201), salvianolic acid B (Lot. ZZS17032203), dihydrotanshinone (Lot. ZZS17032209), cryptotanshinone (Lot. ZZS17032204), and tanshinone IIA (Lot. ZZS17032206) were purchased from* Shanghai ZZBIO Co, *(Shanghai, China). Reference substances of neochlorogenic acid (Lot. X-014-140801), isochlorogenic acid B (Lot. Y-069-141122-1), and isochlorogenic acid C (Lot. Y-070-140801) were purchased from* Chengdu Herbpurify Co.* (Chengdu, China). Reference isochlorogenic acid A (Lot. 20130816) were purchased from* Shanghai Yuanye Bio-Technology Co.* (Shanghai, China). Reference substances of caffeic acid (Lot. 110885-200102), ginsenoside Rg_1_ (Lot. 110703-201027), ginsenoside Re (Lot. 110754-200822), ginsenoside Rb_2_ (Lot. 111715-200802), ginsenoside Rb_3_ (Lot. 111686-201002), 20*S*-ginsenoside F_1_ (Lot. 111763-200601), 20*S*-ginsenoside F_2_ (Lot. 111764-200601), 20*S*-ginsenoside Rh_2_ (Lot. 111748-200501), cinobufagin (Lot. 110803-200605), and resibufogenin (Lot. 110718-200507) were purchased from the National Institutes for Food and Drug Control (Beijing, China). Gamabufotalin, bufotalin, and bufalin were purified from the fresh toad venom (the purity of gamabufotalin was over 92.9% and the purity of bufotalin and bufalin was over 98% based on the area normalization by HPLC analysis). Ginsenoside Rd, chlorogenic acid, rutin, quercetin, protocatechualdehyde, ursodesoxycholic acid, and chenodeoxycholic acid were from the collection of our laboratory and their purities were over 98%. The structures of all reference compounds were shown in Figure 1.

HPLC grade acetonitrile and formic acid (Fisher, Fair Lawn, NJ, USA) and ultrapure water were used. All other chemical reagents were of analytical grade from* Beijing Chemical Corporation* (Beijing, China).

### 2.2. Reference Solution Preparation

Stock solution with a concentration of about 0.1 g·L^−1^ was prepared by dissolving an accurately weighed amount of each reference substance in methanol.

### 2.3. Sample Preparation

#### 2.3.1. XXT Sample

1.0 g (about 2.5 tablets) of pulverized XXT sample was extracted with methanol (25 mL) by ultrasonication (200 w, 53 kHz) for 30 min at room temperature, and the extract was centrifuged for 5 min at 3000 rpm. The supernatant was evaporated at 80°C and the residue was dissolved with aqueous methanol (MeOH-H_2_O, 9:1). The obtained solution was centrifuged for 5 min at 12000 rpm. A volume of 10 *μ*L of the supernatant was used for HPLC analysis.

1 ml of the sample solution for HPLC analysis was diluted to a volume of 10 mL and used for UPLC-ESI-Q-TOF/MS analysis.

#### 2.3.2. Raw Materials

The ethanolic and aqueous extracts of individual herb (Danshen, Maodongqing, Shuizhi, Chuanxiong, and Huaihua) were prepared according to the manufacturing processes of XXT (Figure S1) described in the current Chinese Pharmacopoeia [2]. The ethanolic and aqueous extracts along with Rengong Shexiang, Rengong Niuhuang, Renshen Jingye Zongzaogan, Bingpian, and Chansu were dissolved with aqueous methanol solution (MeOH-H_2_O, 9:1) according to the method described in Section 2.3.1 and used for the comparative analyses on the possible contribution from individual herb to general chromatographic profile of XXT samples.

### 2.4. Qualitative HPLC Analyses of 10 Batches of XXT Samples

The analyses were performed on a Shimadzu HPLC system (Shimadzu, Japan) equipped with an LC-20AT binary pump, a DGU-20A5 degasser, an SIL-20AC autosampler, a CTO-20AC column oven, and an SPD-M20A photodiode array detector. The samples were separated on a Waters XTerra C_18_ column (4.6×250mm, 5*μ*m). The mobile phase consisted of water containing 0.1% acetic acid (A) and acetonitrile (B) using a gradient elution program as follows: 0 min, 10% B; 20 min, 25% B; 30 min, 26% B; 45 min, 30% B; 60 min, 60% B; and 95 min, 80% B. The flow rate was 1.0 mL·min^−1^ and the column temperature was 30°C. The PDA detector recorded UV spectra in the range from 190 nm to 400 nm and HPLC chromatogram was monitored at 251 nm.

### 2.5. UPLC-ESI-Q-TOF/MS Analysis

To comprehensively identify the chemical constituents in XXT samples, a UPLC-ESI-Q-TOF/MS experiment was performed using a Waters Xevo G2-S spectrometer (Waters, America), connected to a Waters UPLC system (Waters, America). The samples were separated on a Waters ACQUITY BEH C_18_ column (2.1×50 mm, 1.7 *μ*m). The mobile phase consisted of water containing 0.1% formic acid (A) and acetonitrile (B) using a gradient elution program as follows: 0 min, 10% B; 4 min, 25% B; 7 min, 25.6% B; 8 min, 36% B; 14 min, 60% B; 18 min, 80% B; and 20 min, 80% B. The mass spectrometer was operated in both positive and negative modes using the following parameters: capillary voltage of 1.8 kV, sample cone voltage of 30 V (ESI+) or 40 V (ESI-), source temperature of 120°C, desolvation temperature of 600°C, and cone gas flow 50 L/h. In MS^E^ mode, the trap collision energy for the low-energy function was set at 6 eV, while the ramp trap collision energy for the high-energy function was set at 25-50 eV. Argon was used as the collision gas for collision-induced dissociation (CID) in MS^E^ and MS^2^ modes. To ensure mass accuracy and reproducibility, the mass spectrometer was calibrated over a range of 100-2000 Da for MS and 50-2000 Da for MS^2^. Leucine-enkephalin (*m/z* 556.2771 in positive ion mode;* m/z* 554.2615 in negative ion mode) was used as an external reference for the LockSpray and was infused at a constant flow of 5 *μ*L·min^−1^. The data were recorded in centroid type during acquisition.

## 3. Results and Discussion

### 3.1. Qualitative Analyses of XXT Samples by HPLC

**Figure 2:**
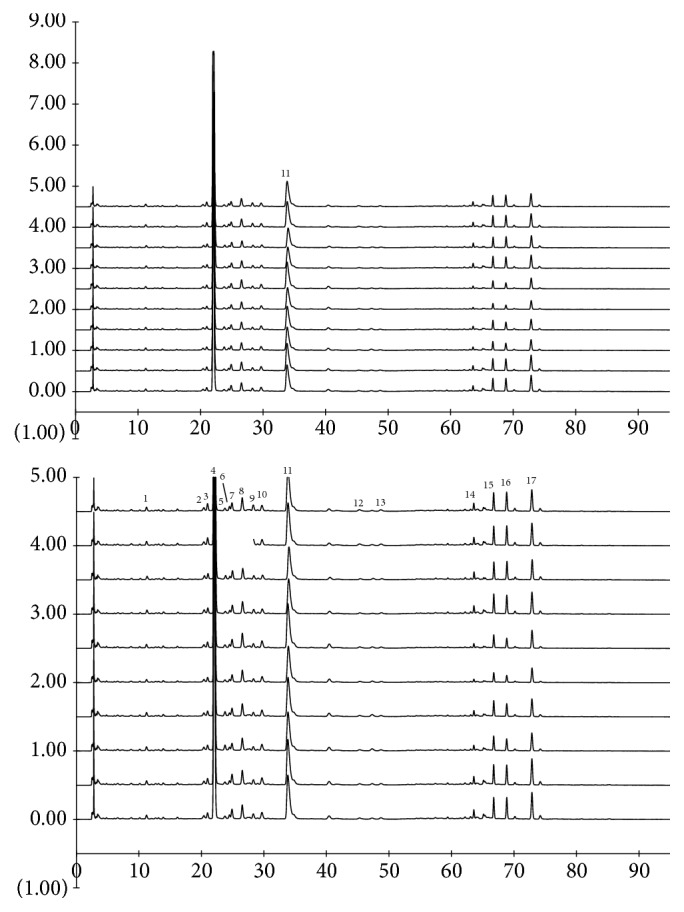
HPLC profiles of Xueshuan Xinmaining Tablet samples at 251 nm (from top to bottom: Lot. 160904, 160905, 161001-161008).

In order to obtain better detection of XXT samples, HPLC conditions were optimized. The acetonitrile-water system displayed more powerful separation ability for the main constituents in all samples than the methanol-water system. When organic acid was added to the mobile phase, the symmetry of most chromatographic peaks was improved. Under optimized conditions, ten batches of XXT samples, together with the reference compounds, were examined and their HPLC chromatograms were shown in Figure 2. High similarity in the number, type, and amount of chemical constituents was observed in the HPLC profile of each individual sample, which suggested the chemical consistency of different batches of XXT samples. The characteristic peak 4 (rutin) represents an important active component in XXT sample with a consistently high content, which was chosen as the reference peak for the calculation of the relative retention time ratio and relative peak area ratio of other common chromatographic peaks. For the validation of the assay procedure, the results of precision and repeatability were indicated by RSDs that were less than 4.9% (n=6) for seventeen peaks (Tables S1~S4). The stability test suggested that these peaks were stable in the sample solution within 24 hr (Tables S5~S6).

To identify the origin of these characteristic peaks from individual raw material, a comparative study was carried out by using various extract of raw materials and XXT samples. Considering the chromatogram recorded at the wavelength of 251 nm, main chromatographic peaks in the HPLC profile were attributed to the following five raw materials: Danshen, Huaihua, Maodongqing, Chuanxiong, and Chansu (Figure S2). The contribution of other raw materials was not manifested, either because of the absence of UV absorption of active compounds or due to the existence of no potent components at the detection condition. More information is expected to further confirm the contribution of each raw material to the general chromatographic profile of XXT sample.

### 3.2. Identification of Chemical Constituents in XXT Sample by UPLC-ESI-Q-TOF/MS

**Figure 3:**
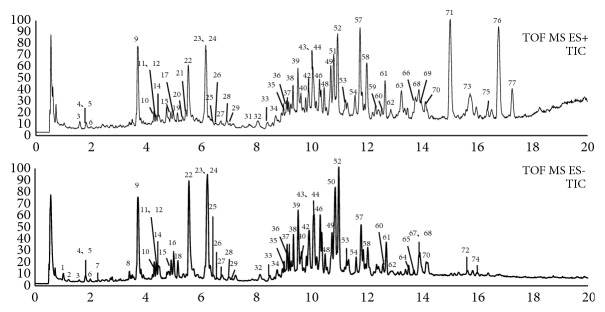
TICs of Xueshuan Xinmaining Tablet in the negative and positive modes.

MS^E^ analyses in the positive ion mode and negative ion mode were selected for obtaining extensive structural information via collision-induced dissociation. For the purpose of determining the optimized CE to generate fragment ions for structural elucidation and characterization, MS/MS experiments at different CE values were performed. Under optimized parameters, the total ion chromatogram of XXT samples are shown in Figure 3.

The fragmentation behaviors of reference compounds were investigated, and these rules were applied for the structural elucidation of their derivatives with the same basic skeleton. A total of 63 compounds in XXT sample were detected and tentatively characterized by comparing the retention times, MS fragmentation behavior, and literature information. Among these compounds, there were twenty saponins, fifteen phenolic acids, ten quinones, eight steroids, four bile acids, four flavonoids, one amino acid, and one lactone. Their chemical structures are provided in Figure 1. For better understanding of the fragmentation behavior, 63 compounds were divided into several groups and deduced as follows. The chromatographic and mass data of the identified constituents were summarized in Table 1.

**Figure 4:**
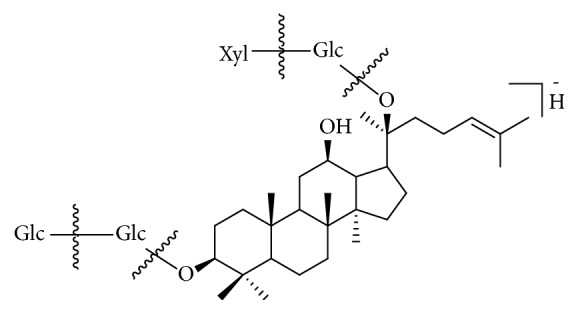
Proposed fragmentation pathway of ginsenoside Rb_3_.

*Saponin Compounds. . *Saponin compounds have no UV absorption, but they could be well detected in both positive and negative ionization modes. Usually, the adduct ions such as [M+Na]^+^ (ESI+) or [M+HCOO]^−^ (ESI-) were detected in MS spectrum. In MS/MS spectrum, a series of fragmentation ions were observed due to the neutral loss of glucose, rhamnose, arabinose, or xylose (see Figure 4). These fragmentation rules are in accordance with the previous data [7, 8]. Totally, 20 ginsenoside saponins were identified, and they are deduced from the raw material, total ginsenoside of ginseng stems and leaves. Among the identified saponins, eight compounds (**23**,** 24**,** 46**,** 47**,** 49**,** 53**,** 61,** and** 74**) were unambiguously identified as ginsenoside Rg_1_, ginsenoside Re, ginsenoside Rb_2_, ginsenoside Rb_3_, 20*S*-ginsenoside F_1_, ginsenoside Rd, 20*S*-ginsenoside F_2_, and 20*S*-ginsenoside Rh_2_. Unknown ginsenoside saponins followed the similar dissociation pathways. For the ginsenosides of protopanaxadiol, the ions at* m/z* 407, 425, and 443 could be found in the positive ion mode. Meanwhile the ginsenosides of protopanaxatriol showed the ions at* m/z *405, 423, and 441 in the positive ion mode.

Ginsenoside Rg_1_ showed [M+Na]^+^ ion at* m/z* 823.4824 (C_42_H_72_O_14_Na) in the positive ion mode and [M+HCOO]^−^ ion at* m/z *845.4895 (C_43_H_73_O_16_) in the negative ion mode. The fragmentation ions at* m/z* 621.4368 and 603.4259 were produced from the protonated ion [M+H]^+^ by losing glucose and the further loss of H_2_O. In the negative MS/MS analysis, [M-H]^−^ at* m/z *799.4838 gave the characteristic ion at* m/z* 637.4307 [M-H-glc]^−^and 475.3773[M-H-2glc]^−^. Compounds** 23** and** 40** showed the quasimolecular ions at* m/z *823 [M+Na]^+^ and 845 [M+HCOO]^−^ in the full mass spectrum. Compound** 23** has the same retention time and MS data as those of reference ginsenoside Rg_1_ and it was identified as ginsenoside Rg_1_. As for compound** 40**, although the fragmentation ions at* m/z* 405, 423, or 441 could not be detected in the MS/MS spectrum, it was temporarily identified as ginsenoside R_f_.

Ginsenoside Re showed [M+Na]^+^ ion at* m/z *969.5427 (C_48_H_82_O_18_Na) in the positive ion mode and [M+HCOO]^−^ ion at* m/z *991.5482 (C_49_H_83_O_20_) in the negative ion mode. The fragmentation ions at* m/z *945.5428 [M-H]^−^, 799.4845 [M-H-rha]^−^, 783.4893 [M-H-glc]^−^, and 637.1430 [M-H-rha-glc]^−^ were found in MS/MS spectrum in the negative ion mode. Compound** 24** showed similar retention time and MS data as those of reference ginsenoside Re and it was identified as ginsenoside Re.

As for reference ginsenoside Rb_2_, [M+Na]^+^ ion at* m/z* 1101.5868 (C_53_H_90_O_22_Na) in the positive ion mode and [M+HCOO]^−^ ion at* m/z* 1123.5908 (C_54_H_91_O_24_) in the negative ion mode are shown in the full mass spectrum. The fragmentation ions at* m/z* 945.5425 [M-H-ara]^−^ and 783.4889 [M-H-ara-glc]^−^ were detected in MS^2^. Reference ginsenoside Rb_3_ (Figure 4) gave the adduct ions at* m/z* 1101.5844 [M+Na]^+^ and 1123.5903 [M+HCOO]^−^. The fragmentation ions at* m/z* 945.5430 [M-H-xyl]^−^, 783.4881 [M-H-xyl-glc]^−^, and* m/z* 621.4352[M-H-xyl-2glc]^−^ were observed in MS^2^.

Compounds** 44**,** 46**, and** 47 **have the same molecular formula of C_53_H_90_O_22_ according to the quasimolecular ions at* m/z* 1101[M+Na]^+^ and 1123 [M+HCOO]^−^. Compounds** 46** and** 47** were unambiguously identified as ginsenoside Rb_2_ and ginsenoside Rb_3_ by comparing the retention time and MS data as those of references. Compound** 44 **was tentatively deduced as ginsenoside Rc.

20*S*-ginsenoside F_1_ gave the adduct ions at* m/z* 661.4300 [M+Na]^+^ (C_36_H_62_O_9_Na) and 683.4375 [M+HCOO]^−^ (C_37_H_63_O_11_Na). In the negative MS/MS experiment, the fragmentation ion at* m/z* 475.3785 [M-H-glc]^−^ was detected. Compound** 49** showed similar retention time and MS data as those of reference 20*S*-ginsenoside F_1_.

Reference ginsenoside Rd showed the adduct ions at* m/z* 969.5379 [M+Na]^+^ and 991.5495 [M+HCOO]^−^ in the full mass spectrum. The fragmentation ions at* m/z *783.4892 [M-H-glc]^−^, 621.4368 [M-H-2glc]^−^, and 459.3842 [M-H-3glc]^−^ were produced by losing glucose. Compound** 53** was identified as ginsenoside Rd by displaying the same retention time and MS information.

As for reference 20*S*-ginsenoside F_2_, it showed the quasimolecular ions at* m/z* 807.4890 [M+Na]^+^ and 829.4949 [M+HCOO]^−^. The deprotonated ion peak at* m/z* 783.4881 [M-H]^−^ produced the fragmentation ion at* m/z* 621.4355 [M-H-glc]^−^ in MS^2^. Compounds** 45**,** 61**,** 64**, and** 65** gave the same adduct ions [M+Na]^+^ in positive MS and [M+HCOO]^−^ in negative MS, indicating the molecular formula as C_42_H_72_O_13_. Compound** 45** was deduced as protopanaxatriol type and the latter three compounds were deduced as protopanaxadiol type on the base of the fragmentation rules described above. Among them, compound** 61** has similar ions in full mass spectrum and MS/MS experiment as those of reference 20*S*-ginsenoside F_2_ and was unambiguously identified as 20*S*-ginsenoside F_2_. The deprotonated ion of** 45** at* m/z* 783.4910 [M-H]^−^ indicated the ion at* m/z* 475.3791 [M-H-rha-glc]^−^, supporting that** 45 **was ginsenoside Rg_2_. Compounds** 64** and** 65** displayed same MS data; however, unfortunately, MS^2^ information could not be detected and they were tentatively identified as 20*R*-ginsenoside Rg_3_ and 20*S*-ginsenoside Rg_3_.

Reference ginsenoside Rh_2_ showed the ions at* m/z* 1245.8995 [2M+H]^+^, 645.4348 [M+Na]^+^, and 667.4437[M+HCOO]^−^. Compound** 74** was identified as 20*S*-ginsenoside Rh_2_ by comparing the retention time and MS data.

Compounds** 35**,** 36**, and** 37 **have the same molecular formula of C_48_H_82_O_19_ and were tentatively characterized as 20*S*-glc-ginsenoside R_f_, beads ginseng saponin F_1_, and notoginsenoside R_3_. They gave [M+Na]^+^ ions at* m/z* 985.5332, 985.5319, and 985.5339 in the positive ion mode and [M+HCOO]^−^ ions at* m/z* 1007.5457, 1007.5457, and 1007.5459 in the negative ion mode. In their MS/MS experiments, the fragmentation ions of** 35** at* m/z* 799.4843 and 637.4273 were produced from the ion at m/z 961.5391 [M-H]^−^ by loss of glucose unit. Similarly, the fragmentation ions of** 36 **and** 37** at* m/z* 799 and 637 were yielded. The retention order of three chromatographic peaks is determined according to the description in the literature [9].

The chemical formulas of compounds** 42** and** 43 **are calculated as C_41_H_70_O_13_ based on the ions at* m/z* 793.4705 [M+Na]^+^ in the positive ion mode and 815.4818 [M+HCOO]^−^ in the negative ion mode. Both compounds showed the fragmentation ions at* m/z *405, 423, or 441 indicating that they are due to protopanaxatriol type. In MS/MS analysis, the ions at* m/z* 637.4326 [M-H-xyl]^−^ and 475.3794 [M-H-xyl-glc]^−^ for compound** 42**, as well as 637.4319 [M-H-ara]^−^ and 475.3788 [M-H-ara-glc]^−^ for compound** 43**, were detected from the deprotonated ions at* m/z* 769.4753 [M-H]^−^ and 769.4758 [M-H]^−^, respectively. Compounds** 42** and** 43** were identified as notoginsenoside R_2_ and ginsenoside F_3_.

Compound** 54** showed the quasimolecular ions at* m/z* 939.5284 [M+Na]^+^ and 961.5390 [M+HCOO]^−^, indicating the molecular formula as C_47_H_80_O_17_. The fragmentation ions 783.4901 [M-H-xyl]^−^ and 621.4363 [M-H-xyl-glc]^−^ were detected, supporting that** 54 **was gypenoside IX.

Compound** 60** showed the quasimolecular ions at* m/z* 789.4742 [M+Na]^+^ and 811.4871 [M+HCOO]^−^ and the molecular formula was calculated as C_42_H_70_O_12_. In the MS/MS analysis, the deprotonated ion at* m/z *765.4792[M-H]^−^ gave the ions at* m/z *603.4261 [M-H-glc]^−^ and 441.3730 [M-H-2glc]^−^. Further loss of H_2_O yielded the ions at* m/z *423 and 405. It was identified as ginsenoside Rk_1_.

Compound** 34** showed [M+Na]^+^ ion at* m/z* 1117.5740 and [M+HCOO]^−^ ion at* m/z* 1139.5891, suggesting the molecule formula of C_53_H_90_O_23_. Besides, it showed [M-H]^−^ ion at* m/z* 1093.5826 and fragment ions at* m/z* 961 [M-H- xyl/ara]^−^, 799 [M-H-xyl/ara-glc]^−^, and 637 [M-H-xyl/ara-2glc]^−^, concerning consecutive loss of 132, 294, and 456. These data were in accordance with those of floral ginsenoside P, floranotoginsenoside D, notoginsenoside FT_3_, or yesanchinoside H [9], but its structure was not accurately determined based on the limited information.

Compound** 72** showed [M+Na]^+^ ion at m/z 789.4742 and [M+HCOO]^−^ ion at m/z 811.4847, indicating the molecule formula as C_42_H_70_O_12._ Besides, the ions at* m/z* 603.4251 [M-H-162]^−^ and 471.3466 [M-H-162-132]^−^ in the MS/MS analysis suggested that the glycoside chain of** 72** consisted of a molecule of glucose and a molecule of arabinose or xylose. It could be one of ilexoside A or ilexoside D described in the literature [10].

*Flavonoids*. Flavonoids always present high sensitivity in the UV spectra. They could be well detected in both positive and negative ionization modes. Rutin, which has UV *λ*_max_ absorption at 255 and 353 nm, showed the protonated ion at* m/z *611.1656 [M+H]^+^ and deprotonated ion at* m/z* 609.1461 [M-H]^−^ as well as the ion at* m/z* 1219.3008 [2M-H]^−^. Quercetin, with UV *λ*_max_ absorption at 253 and 356 nm, displayed the deprotonated ion at* m/z* 301.0376 [M-H]^−^. Four compounds (**9**,** 10**,** 13**, and** 26**) were determined as flavonoid derivatives due to the typical UV absorption. Especially, compound** 9** was one of main constituents of XXT. Compounds** 9** and** 26 **were unambiguously identified as rutin and quercetin based on the direct comparison of their UV spectra, and mass spectra with those of the authentic compounds. Compound** 10** has UV *λ*_max_ absorption at 264 and 343 nm. The molecular formula was calculated as C_27_H_30_O_15_ by the quasimolecular ion at* m/z* 595.1652 [M+H]^+^, 617.1472 [M+Na]^+^, and 593.1505 [M-H]^−^. Other fragment ions at* m/z* 449.1083 [M+H-rha]^+^ and 287.1055 [M+H-rha-glc]^+^ were detected. Therefore, compound** 10 **was identified as kaempferol-3-*O*-rutinoside. Compound** 13** has UV *λ*_max_ absorption at 252 and 339 nm. The molecular formula was calculated as C_28_H_32_O_16_ by the quasimolecular ion at* m/z* 625.1754 [M+H]^+^, 647.1578 [M+Na]^+^, and 623.1616 [M-H]^−^, suggesting it was isorhamnetin 3-*O*-rutinoside [11].

*Phenolic acids.* Phenolic acids are the main constituents of XXT. This category of compounds was primarily derived from the ingredient herb,* Salviae miltiorrhizae radix et rhizoma* and* Hairy holly root*. In this study, 14 phenolic acids were found, among which 10 compounds (**1**,** 2**,** 4**,** 5**,** 7**,** 11**,** 14**,** 15**,** 16**, and** 22**) were identified as danshensu, neochlorogenic acid, chlorogenic acid, protocatechuic aldehyde, caffeic acid, isochlorogenic acid A, isochlorogenic acid B, isochlorogenic acid C, rosmarinic acid, and salvianolic acid B by using reference substances. Other compounds (**12**,** 17**,** 18**, and** 32**) were tentatively characterized by comparing UV absorption, retention time, molecular formula, and mass fragmentation pathways [12–14].

Danshensu has UV *λ*_max_ absorption at 279 nm and retention time at 1.03 min. It showed the precursor ion at* m/z* 197.0443 and the molecular formula was calculated as C_9_H_10_O_5_. In the full mass spectrum, the [2M-H]^−^ ion at* m/z* 395.07949 was found. The MS/MS spectrum of [M-H]^−^ exhibited an obvious fragment ion, [M-H-H_2_O]^−^ at* m/z *179.0338, and further loss of COOH obtained ion at* m/z *135.0438. Compound** 1** was identified as danshensu.

Neochlorogenic acid has UV *λ*_max_ absorption at 323 nm. In the full mass spectrum, the deprotonated ion at* m/z* 353.0869 [M-H]^−^ was found. The MS/MS spectrum of [M-H]^−^ showed ions at* m/z* 191.0548, 179.0336, and 135.0438. Compound** 2** was identified as neochlorogenic acid.

Chlorogenic acid has UV *λ*_max_ absorption at 325 nm and showed the quasimolecular ion at* m/z *353.0872 [M-H]^−^ and 707.1482 [2M-H]^−^. The fragment ion at* m/z* 191.0553 was yielded by losing C_9_H_6_O_3_. Compound** 4 **was identified as chlorogenic acid.

Protocatechuic aldehyde has UV *λ*_max_ absorption at 229, 278, and 310 nm. The [M-H]^−^ ion at* m/z* 137.0236 was detected. In the MS/MS analysis, the ion at* m/z* 108.0204 was yielded by losing CHO and further losing oxygen produced from the ion at* m/z* 92.0256. Compound** 5** was identified as protocatechuic aldehyde.

Caffeic acid has UV *λ*_max_ absorption at 238 and 322 nm. It showed [M-H]^−^ ion at* m/z* 179.0339 in the full mass spectrum and the ion at* m/z *135.0438 by losing CO_2_ in the MS/MS experiment. Compound** 7** was identified as caffeic acid.

The parent ions of three compounds (**11**,** 14**, and** 15**) at* m/z *515 [M-H]^−^ and 517 [M+H]^+^ were easily located in the chromatogram of XXT, suggesting the molecular formula of C_25_H_24_O_12_. They were assigned as dicaffeoylquinic acids by comparison with retention times. Besides, they gave the same fragment ions with those of chlorogenic acid, such as m/z 191, 179, 173, and 135. in MS/MS experiments. The fragmentation pathways are in accordance with those described in the literature [15]. Therefore, compounds** 11**,** 14**, and** 15 **were isochlorogenic acid B, A, and C.

Rosmarinic acid included a caffeic acid moiety and a molecule of danshensu. It showed [M-H]^−^ ion at* m/z *359.0759. The fragment ions at* m/z* 197.040 and 179.0332 were yielded by losing caffeic acid or danshensu moiety in MS^2^. Besides, the ion at* m/z *161.0231 was yielded by further loss of H_2_O. Compound** 16** was identified as rosmarinic acid.

Salvianolic acid B showed [M-H]^−^ ion at* m/z* 717.1464. Besides, the fragment ions at* m/z* 519.0934 ([M-H-C_9_H_10_O_5_]^−^), 339.0504, and 321.0392 were detected in the MS/MS analysis. The characteristic fragmentation pathway of losing C_9_H_10_O_5_ is in accordance with those described in the literature [16]. Based on the retention time and the mass fragmentation pathway, compound** 22 **was identified as salvianolic acid B.

Compound** 12** has UV *λ*_max_ absorption at 244 and 327 nm. The molecular formula was calculated as C_27_H_22_O_12_ on the basis of the quasimolecular ions at* m/z* 539.1149 [M+H]^+^ and 537.1031 [M-H]^−^. In the MS/MS experiment, it indicated the characteristic ion at* m/z* 339.0500 [M-H-C_9_H_10_O_5_]^−^, supporting that compound** 12** was salvianolic acid H.

Compound** 17** has UV *λ*_max_ absorption at 250 and 306 nm. It showed the ion at* m/z *341.0666 [M+H]^+^. In the MS/MS analysis, the ions at* m/z *295.0617 and 279.0659 were yielded by losing CH_2_O_2_ and CH_2_O_3_. Compound** 17** was identified as salvianolic acid G.

Compound** 18** has UV *λ*_max_ absorption at 251 and 317 nm. It showed [M-H]^−^ ion at* m/z* 493.1134 and the molecular formula was calculated as C_26_H_22_O_10_. In the MS/MS analysis, the fragment ion at* m/z* 295.0604 [M-H-C_9_H_10_O_5_]^−^ was detected, suggesting compound** 18 **was salvianolic acid A.

Compound** 32** showed the ions at* m/z *567.1498 [M+H]^+^, 589.1320 [M+Na]^+^, and 565.1342 [M-H]^−^ in MS spectrum, revealing molecular formula of C_29_H_26_O_12_. Besides, fragment ion at* m/z *369.0971 [M-H-C_9_H_10_O_5_]^−^ was observed. Compound** 32 **was identified as ethyl lithospermate.

**Figure 5:**
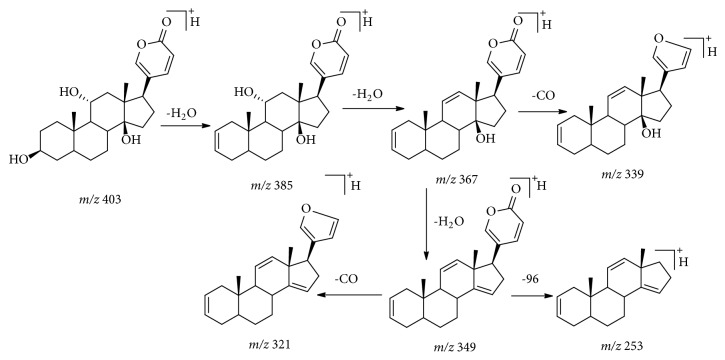
Proposed fragmentation pathway of gamabufotalin.

*Bufadienolides.* The MS/MS behaviors of bufadienolides have been extensively described [17, 18]. Briefly, for bufadienolides with only hydroxyl substituents, the fragmentation was characterized by successive eliminations of H_2_O and CO molecules, and the profile of MS/MS product ions was correlated with the number of hydroxyl groups. If a C-16 acetoxyl group was present, the fragmentation of [M+H]^+^ ions was triggered by initial loss of 60 Da (HOAc). The elimination of CO was significant for bufadienolides with a 19-formyl group, and the 19-hydroxyl group could be characterized by the loss of 30 Da (HCHO). These fragmentation rules were applied to the identification of bufadienolides in XXT sample. As shown in Table 1, eight bufadienolides were screened from XXT, six of which were unambiguously identified as gamabufotalin (**19**), arenobufagin (**25**), bufotalin (**41**), bufalin (**50**), resibufogenin (**55**), and cinobufagin (**56**) by comparison with reference substances isolated from toad venom. The other two bufadienolides were tentatively identified as bufarenogin (**20**) and hellebrigenin (**29**) [18, 19]. Taking gamabufotalin as a case, the fragmentation rules of bufadienolides were explained (Figure 5). Gamabufotalin showed UV *λ*_max_ absorption at 295 nm. In its full mass spectrum, [M+H]^+^ ion at* m/z* 403.2486 was found and the fragment ions at* m/z* 385.2366 [M+H-H_2_O]^+^, 367.2262 [M+H-2H_2_O]^+^, and 349.2169 [M+H-3H_2_O]^+^ were detected in MS^2^.

*Bile acids.* Bile acid derivatives are in lack of conjugated system and their UV absorption is not obvious. However, they always present high sensitivity in the negative ion mode. The deprotonated ion [M-H]^−^ and adduct ion [M+HCOO]^−^ were obviously detected in MS spectrum. The loss of side chain was commonly observed in MS^2^. Four bile acid derivatives were detected from XXT sample, two of which were unambiguously identified as ursodeoxycholic acid (**58**, UDCA) and chenodeoxycholic acid (**70**, CDCA) based on the direct comparison of reference substances. The other two compounds were tentatively identified as cholanic acid (**57**) and hyodeoxycholic acid (**67**). They are derived from the raw material, Rengong Niuhuang (*Bovis calculus artifactus*) [20].

Ursodeoxycholic acid showed the ions at* m/z *391.2851 [M-H]^−^, 437.2916 [M+HCOO]^−^, and 783.5778 [2M-H]^−^ in the negative ion mode. In the positive ion mode, the ion at* m/z *357.2807 [M+H-2H_2_O]^+^ was assigned as the loss of two molecules of H_2_O. The ion at* m/z *321.2587 represented its side chain loss in MS^2^. Compound** 58** was identified as UDCA.

CDCA showed the ions at* m/z* 391.2872 [M-H]^−^, 437.2907[M+HCOO]^−^, and 783.5798 [2M-H]^−^ in the negative ion mode. In the positive ion mode, the loss of H_2_O unit yielded [M+H-2H_2_O]^+^ ion at* m*/*z *357.2809. The occurrence of the ion at* m/z *321.2586 in MS^2^ was due to side chain loss. Compound** 70** was identified as CDCA.

Compound** 57** showed [M+Na]^+^ ion at* m/z *431.2778 in positive mode, and [M-H]^−^ ion at* m/z* 407.2803, [M+HCOO]^−^ ion at* m/z *453.2857, and [2M-H]^−^ ion at* m/z *815.5698 in the negative mode. The ions representing a series of H_2_O loss was observed, such as 373.2749 [M+H-2H_2_O]^+^ and 355.2649 [M+H-3H_2_O]^+^. It was tentatively characterized as cholanic acid.

Compound** 67 **showed [M-H]^−^ ion at* m/z *391.2852, [M+HCOO]^−^ ion at* m/z* 437.2903, and [2M-H]^−^ at* m/z *783.5792 in the full mass spectrum. The molecular formula was calculated as C_24_H_40_O_4_. It was tentatively deduced as hyodeoxycholic acid.

**Figure 6:**
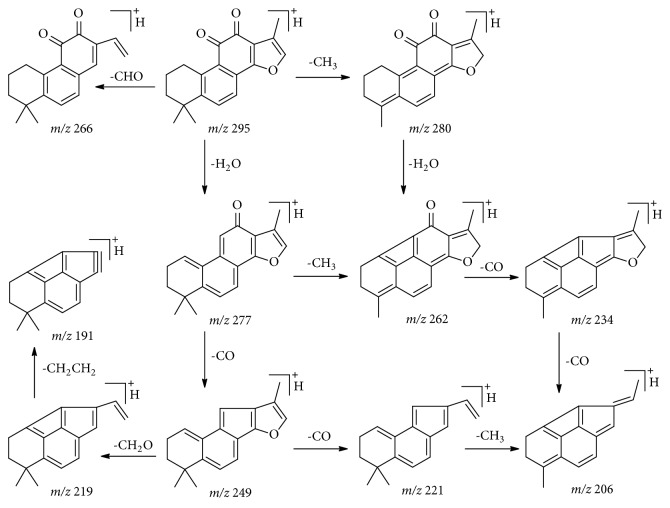
Proposed fragmentation pathway of tanshinone IIA.

*Quinones derivatives.* Quinones derivatives are another kind of active constituents from Danshen and they were easily detected in XXT sample. Compounds** 63**,** 71**, and** 76 **were identified as dihydrotanshinone, cryptotanshinone, and tanshinone II A by comparison with reference substances. They displayed similar fragmentation pathways concerning successive eliminations of H_2_O and CO molecules. Tanshinone IIA was used as an example to illustrate the fragmentation pathway of quinones constituents as shown in Figure 6. Based on these rules as well as empirical molecular formula, 7 compounds were characterized as tanshindiol A** 31**, 1-oxo tanshinone IIA** 59**, neotanshinone D** 62**, tetrahydrotanshinone I** 66**, methyltanshinonate** 69**, methylenetanshinquinone** 73**, and miltirone** 77**. [12, 16, 20, 21].

*Phthalide derivative.* Compound** 21** has UV absorption at 276 nm. Its molecular formula was calculated as C_12_H_14_O_3_ on the basis of [M+H]^+^ ion at* m/z* 207.1024. The above data were in accordance with those of 4-hydroxyl-3-butylphthalide in the literature [22]. Compound** 21** was tentatively assigned as 4-hydroxyl-3-butylphthalide. It could be derived from individual herb* Chuanxiong rhizome* [23].

Among the identified compounds, most constituents were derived from the raw materials, Danshen and Renshen Jingye Zongzaogan, and a small proportion of compounds were considered from Huaihua, Maodongqing, Chuanxiong, Chansu, and Rengong Niuhuang. The other ingredients, Rengong Shexiang, Bingpian, and Shuizhi, were not characterized in the present HPLC and UPLC-QTOF/MS condition. This could be related to the prescription of raw materials and the manufacturing process employed. Usually, muscone, one of active constituents in Rengong Shexiang and borneol are detected by GC or GC-MS [3]. They are not easily detected in the liquid chromatography. Additionally, it is worth noting that no chemical information of Shuizhi, a famous traditional Chinese medicine originated from animal source, was found, although small molecule compounds with diverse structures have been reported from this drug [24]. More effort is made to explain its prescription role in XXT product.

## 4. Conclusion

In this work, HPLC analysis was employed to find out the common chromatographic peak in various batches of XXT samples and UPLC-Q-TOF/MS was used for the identification of main constituents in the typical XXT sample. As a result, a total of 63 constituents including twenty saponins, four flavonoids, fifteen phenolic acids, eight steroids, four bile acids, ten quinones, and other two compounds were identified or tentatively characterized based on the comparison of retention time and UV spectra with authentic compounds as well as by summarized MS fragmentation rules and matching empirical molecular formula with those of published components. The present investigation clearly understood the nonvolatile constituents in XXT and provided good basis for further study on the active substances and quality control of this preparation.

## Figures and Tables

**Table 1:** Retention time (*t*_R_), UV, and MS data of the identified compounds in XXT samples.

No.	t_R_/min	UV	Identified compounds	Formula	[M+H]^+^/[M+Na]^+^	Calc.mass	Δppm	[M-H]^−^	Calc.mass	Δppm	[M+HCOO]^−^/[2M-H]^−^	Calc.mass	Δppm	Source^a^
		*λ*_max_/nm												
1	1.03	282	danshensu	C_9_H_10_O_5_				197.0446	197.0450	-2.5	/395.0974	395.0978	-1.0	DS
2	1.22	295	neochlorogenic acid	C_16_H_18_O_9_				353.0869	353.0873	-1.1				DS/MDQ
3	1.59		suberyl arginine	C_14_H_26_O_5_N_4_	331.1977/	331.1981	-1.2	329.1819	329.1825	-1.8				CS
4	1.83	320	chlorogenic acid	C_16_H_18_O_9_	355.1026/	355.1029	-0.8	353.0876	353.0873	0.8	/707.1840	707.1823	2.4	DS/MDQ
5	1.83	220, 318	protocatechuic aldehyde	C_7_H_6_O_3_				137.0235/	137.0239	-2.9				DS
6	1.99	233	cryptochlorogenic acid		/377.0854			353.0871						DS/MDQ
7	2.28	232, 318	caffeic acid	C_9_H_8_O_4_				179.0341	179.0344	-1.7				DS
8	3.41	244, 329	unidentified		/465.1384			441.1395						
9	3.68	254, 352	rutin	C_27_H_30_O_16_	611.1618/	611.1612	1.0	609.1458	609.1456	0.3	/1219.3020	1219.2989	2.5	HH
10	4.31	264, 343	kaempferol-3-O-	C_27_H_30_O_15_	595.1652/	595.1663	-1.8	593.1505	593.1506	-0.1				HH
			rutinoside		/617.1472	617.1482	-1.6							
11	4.35	244, 327	isochlorogenic acid B	C_25_H_24_O_12_	517.1331/	517.1346	-2.9	515.1188	515.1190	-0.4				DS/MDQ
					/539.1177	539.1165	2.2							
12	4.39	244, 327	salvianolic acid H	C_27_H_22_O_12_	539.1149/	539.1190	-7.6	537.1031	537.1033	-0.4				DS
13	4.42	252, 339	isorhamnetin 3-O-rutinoside	C_28_H_32_O_16_	625.1754/	625.1769	-2.4	623.1616	623.1612	0.6				HH
					/647.1578	647.1588	-1.5							
14	4.50	329	isochlorogenic acid A	C_25_H_24_O_12_	/539.1154	539.1165	-2.0	515.1193	515.1190	0.6				DS/MDQ
15	4.90	244, 326	isochlorogenic acid C	C_25_H_24_O_12_	517.1359/	517.1346	2.5	515.1192	515.1190	0.4				DS/MDQ
					/539.1176	539.1165	2.0							
16	5.02	244, 329	rosmarinic acid	C_18_H_16_O_8_				359.0766	359.0767	-0.3	/719.1610	719.1612	-0.3	DS
17	5.13	250, 306	salvianolic acid G	C_18_H_12_O_7_	341.0666/	341.0661	1.5							DS
18	5.17	251, 317	salvianolic acid A	C_26_H_22_O_10_				493.1134	493.1135	0.2				DS
19	5.28		gamabufotalin	C_24_H_34_O_5_	403.2483/	403.2484	-0.2				447.2381/	447.2383	-0.4	CS
20	5.32		bufarenogin	C_24_H_32_O_6_	417.2273/	417.2277	-1.0							CS
21	5.43	276	4-hydroxyl-3-butylphthalide	C_12_H_14_O_3_	207.1024/	207.1021	1.4							CX
22	5.53	232	salvianolic acid B	C_36_H_30_O_16_				717.1475	717.1456	2.6				DS
23	6.17		ginsenoside Rg_1_	C_42_H_72_O_14_	/823.4792	823.4820	-3.4				845.4905/	845.4899	0.7	RSZZG
24	6.24		ginsenoside Re	C_48_H_82_O_18_	/969.5378	969.5399	-2.2	945.5450	945.5423	2.9	991.5548/	991.5478	7.1	RSZZG
25	6.44		arenobufagin	C_24_H_32_O_6_	417.2271/	417.2277	-1.4	415.2111	415.2121	-2.4	461.2168/	461.2175	-1.5	CS
26	6.45		quercetin	C_15_H_10_O_7_	303.0506/	303.0505	0.3	301.0346	301.0348	-0.7				HH
27	6.71		unidentified		765.2371									
28	6.99		unidentified		/677.4221						699.4323/			
29	7.17	245, 328	hellebrigenin	C_24_H_32_O_6_	417.2274	417.2277	-0.7				461.2173/	461.2175		CS
30	7.25		unidentified					677.1508						
31	7.49		tanshindiol A	C_18_H_16_O_5_	313.1079/	313.1076	1.0							DS
					/335.0901	335.0895	1.8							
32	8.14		ethyl lithospermate	C_29_H_26_O_12_	567.1498/	567.1503	-0.9	565.1342	565.1346	-0.1				DS
					/589.1320	589.1322	-0.3							
33	8.49		unidentified		/755.1589			731.1622						
34	8.65		unidentified		/1117.5740						1139.5891/			
35	9.01		20*S*-glc-ginsenoside Rf	C_48_H_82_O_19_	/985.5332	985.5348	-1.6				1007.5457/	1007.5427	3.0	RSZZG
36	9.15		beads ginseng saponins F_1_	C_48_H_82_O_19_	/985.5319	985.5348	-2.9				1007.5457/	1007.5427	3.0	RSZZG
37	9.26		notoginsenoside R_3_	C_48_H_82_O_19_	/985.5339	985.5348	-0.9				1007.5459/	1007.5427	3.2	RSZZG
38	9.35		unidentified		/951.4901						973.5042/			
39	9.52		unidentified		/538.2797			514.2904			/1029.5789			
40	9.64		ginsenoside Rf	C_42_H_72_O_14_	801.4985/	801.5000	-1.9				845.4923/	845.4899	2.8	RSZZG
					/823.4802	823.4820	-2.2							
41	9.72		bufotalin	C_26_H_36_O_6_	445.2588/	445.2590	-0.4				489.2489/	489.2488	0.2	CS
42	9.93		notoginsenoside R_2_	C_41_H_70_O_13_	/793.4705	793.4714	-1.1				815.4818/	815.4793	2.5	RSZZG
43	10.07		ginsenoside F_3_	C_41_H_70_O_13_	/793.4700	793.4714	-1.8				815.4820/	815.4793	3.3	RSZZG
44	10.10		ginsenoside Rc	C_53_H_90_O_22_	/1101.5831	1101.5821	0.9				1123.5947/	1123.5900	4.2	RSZZG
45	10.20		ginsenoside Rg_2_	C_42_H_72_O_13_	/807.4908	807.4871	4.3				829.4968/	829.4949	2.3	RSZZG
46	10.31		ginsenoside Rb_2_	C_53_H_90_O_22_	/1101.5798	1101.5821	-2.1				1123.5952/	1123.5900	4.6	RSZZG
47	10.38		ginsenoside Rb_3_	C_53_H_90_O_22_	/1101.5797	1101.5821	-2.2				1123.5940/	1123.5900	3.6	RSZZG
48	10.49		unidentified		466.3162/			464.3032						
49	10.75		20*S*-ginsenoside F_1_	C_36_H_62_O_9_	/661.4288	661.4292	-0.6				683.4393/	683.4370	3.4	RSZZG
50	10.73		bufalin	C_24_H_34_O_4_	387.2531/	387.2535	-1.0							CS
51	10.86		unidentified		/969.5361	969.5399	-3.9				991.5539/	991.5478	6.2	
52	10.99		unidentified		503.3368/			501.3224						
53	11.27		ginsenoside Rd	C_48_H_82_O_18_	/969.5374	969.5399	-2.6				991.5509/	991.5478	3.1	RSZZG
54	11.61		gypenoside IX	C_47_H_80_O_17_	/939.5284	939.5293	-1.0				961.5390/	961.5372	1.9	RSZZG
55	11.68		resibufogenin	C_24_H_32_O_4_	385.2385/	385.2379	1.6							CS
56	11.71		cinobufagin	C_26_H_34_O_6_	443.2435/	443.2434	0.2							CS
57	11.79		cholic acid	C_24_H_40_O_5_	/431.2778	431.2773	1.2	407.2803	407.2797	1.5	453.2857/	453.2852	1.1	RGNH
											/815.5698	815.5673	3.1	
58	12.03		ursodeoxycholic acid	C_24_H_40_O_4_				391.2851	391.2848	0.8	437.2902/	437.2903	1.4	RGNH
											/783.5796	783.5775	2.6	
											/783.5778	783.5775	0.4	
59	12.41		1-oxo tanshinone IIA	C_19_H_16_O_4_	309.1134/	309.1127	2.3							DS
60	12.59		ginsenoside Rk_1_	C_42_H_70_O_12_	/789.4742	789.4765	-2.9				811.4871/	811.4844	3.3	RSZZG
61	12.66		20*S*-ginsenoside F_2_	C_42_H_72_O_13_	/807.4860	807.4871	-1.4				829.4964/	829.4949	1.8	RSZZG
62	12.89		neotanshinone D	C_21_H_20_O_4_	337.1431/	337.1440	-2.7							DS
63	13.25		dihydrotanshinone	C_18_H_14_O_3_	279.1033/	279.1021	4.3							DS
					/301.0849	301.0841	2.7							
64	13.52		20*R*-ginsenoside Rg_3_	C_42_H_72_O_13_	/807.4858	807.4871	-2.1				829.4963/	829.4949	1.7	RSZZG
65	13.71		20*S*-ginsenoside Rg_3_		/807.4852	807.4871	-2.4				829.4968/	829.4949	2.3	RSZZG
66	13.76		tetrahydro-tanshinone I	C_18_H_16_O_3_	281.1189/	281.1178	3.9							DS
					/303.1004	303.0997	2.3							
67	13.85		hyodeoxycholic acid	C_24_H_40_O_4_				391.2852	391.2848	1.0	437.2903/	437.2903	0.0	RGNH
											/783.5792	783.5775	2.2	
68	13.92		unidentified		503.3368/			501.3222			/1003.6544			
69	13.98		methyl tanshinonate	C_20_H_18_O_5_	339.1240/	339.1232	2.4							DS
					/361.1054	361.1052	1.4							
70	14.15		chenodeoxycholic acid	C_24_H_40_O_4_				391.2848	391.2848	0.0	437.2901/	437.2903	-0.5	RGNH
											/783.5789	783.5775	1.8	
71	15.05		cryptotanshinone	C_19_H_20_O_3_	297.1508/	297.1491	5.7							DS
					/319.1313	319.1310	0.9							
72	15.62		ilexoside A/D	C_42_H_70_O_12_	/789.4742	789.4765	-2.9				811.4847/	811.4844	0.4	MDQ
73	15.69	293	methylene	C_18_H_14_O_3_	279.1030/	279.1021	3.2							DS
			tanshinquinone		/301.0848	301.0841	2.3							
74	16.03		20*S*-ginsenoside Rh_2_	C_36_H_62_O_8_							667.4425/	667.4421	0.6	RSZZG
75	16.39		unidentified		496.3405/						540.3302/			
76	16.81	267	tanshinone IIA	C_19_H_18_O_3_	295.1361/	295.1334	9.1							DS
					/317.1165	317.1154	3.5							
77	17.31		miltirone	C_19_H_22_O_2_	283.1709/	283.1698	3.9							DS
					/305.1525	305.1517	2.6							

^a^ Danshen, Huaihua, Maodongqing, Chansu, Chuanxiong, Renshen Jingye Zongzaogan, and Rengong Niuhuang are abbreviated as DS, HH, MDQ, CS, CX, RSZZG, and RGNH, respectively.

## Data Availability

HPLC and UPLC-ESI-Q-TOF/MS data used to support the findings of this study are available from the corresponding author upon request.

## Supplementary Materials

Supplementary Materials The pharmaceutical manufacture process of XXT described in current Chinese Pharmacopoeia is shown in Figure S1. HPLC of XXT sample and extract of each raw material at 251 nm are shown Figure S2. Relative retention time ratio and relative area ratio of common characteristic peaks in precision, repeatability, and stability test for the HPLC method validation are shown in Tables S1~S6.
