# Supplementary material for: Chemical Profiling of Xueshuan Xinmaining Tablet by HPLC and UPLC-ESI-Q-TOF/MS
Source: Evid Based Complement Alternat Med. 2018 Oct 21;2018:2781597. doi: 10.1155/2018/2781597 (PMC6215575; doi:10.1155/2018/2781597)
Supplement: Supplementary Materials — The pharmaceutical manufacture process of XXT described in current Chinese Pharmacopoeia is shown in Figure S1. HPLC of XXT sample and extract of each raw material at 251 nm are shown Figure S2. Relative retention time ratio and relative area ratio of common characteristic peaks in precision, repeatability, and stability test for the HPLC method validation are shown in Tables S1~S6. [file 2781597.f1.zip › 2781597.f1/Figure S1_ECAM_2499613.pptx]

## Slide 1
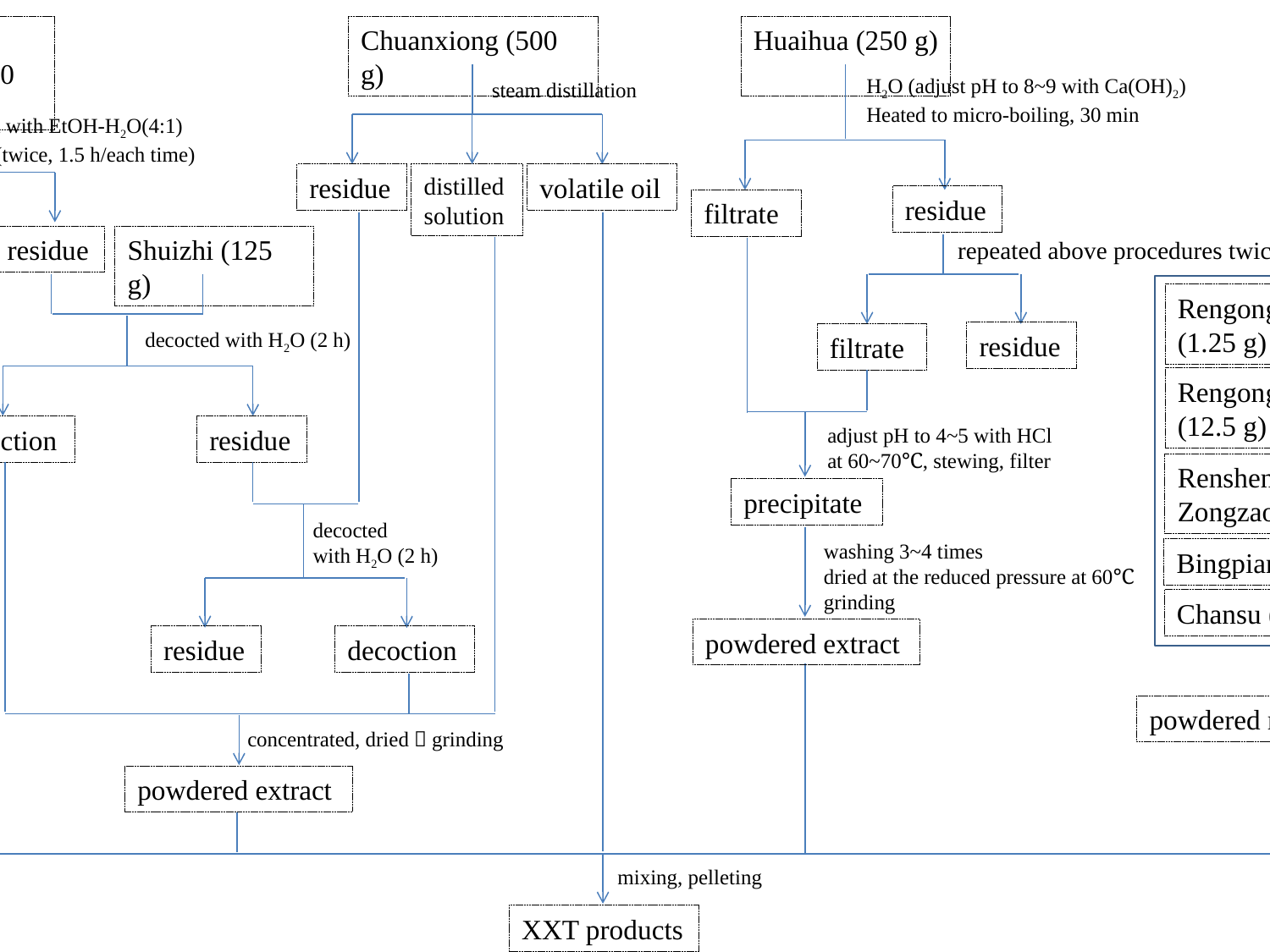

Chuanxiong (500 g)
Danshen (500 g)
Maodongqing (250 g)
Huaihua (250 g)
H2O (adjust pH to 8~9 with Ca(OH)2)
Heated to micro-boiling, 30 min
steam distillation
Extract with EtOH-H2O(4:1)
reflux (twice, 1.5 h/each time)
residue
distilled solution
volatile oil
residue
filtrate
residue
Shuizhi (125 g)
ethanolic solution
repeated above procedures twice
Rengong Shexiang (1.25 g)
Rengong Niuhuang (12.5 g)
Renshen Jingye Zongzaogan (25 g)
Bingpian (2.5 g)
Chansu (1.25 g)
concentrated, dried
grinding
decocted with H2O (2 h)
residue
filtrate
powdered extract
adjust pH to 4~5 with HCl at 60~70℃, stewing, filter
decoction
residue
precipitate
decocted
with H2O (2 h)
washing 3~4 times
dried at the reduced pressure at 60℃
grinding
powdered extract
residue
decoction
grinding
powdered raw materials
concentrated, dried，grinding
powdered extract
mixing, pelleting
XXT products
