# Supplementary material for: Chemical Profiling of Xueshuan Xinmaining Tablet by HPLC and UPLC-ESI-Q-TOF/MS
Source: Evid Based Complement Alternat Med. 2018 Oct 21;2018:2781597. doi: 10.1155/2018/2781597 (PMC6215575; doi:10.1155/2018/2781597)
Supplement: Supplementary Materials — The pharmaceutical manufacture process of XXT described in current Chinese Pharmacopoeia is shown in Figure S1. HPLC of XXT sample and extract of each raw material at 251 nm are shown Figure S2. Relative retention time ratio and relative area ratio of common characteristic peaks in precision, repeatability, and stability test for the HPLC method validation are shown in Tables S1~S6. [file 2781597.f1.zip › 2781597.f1/FIGURE S2_ECAM_2499614.pptx]

## Slide 1
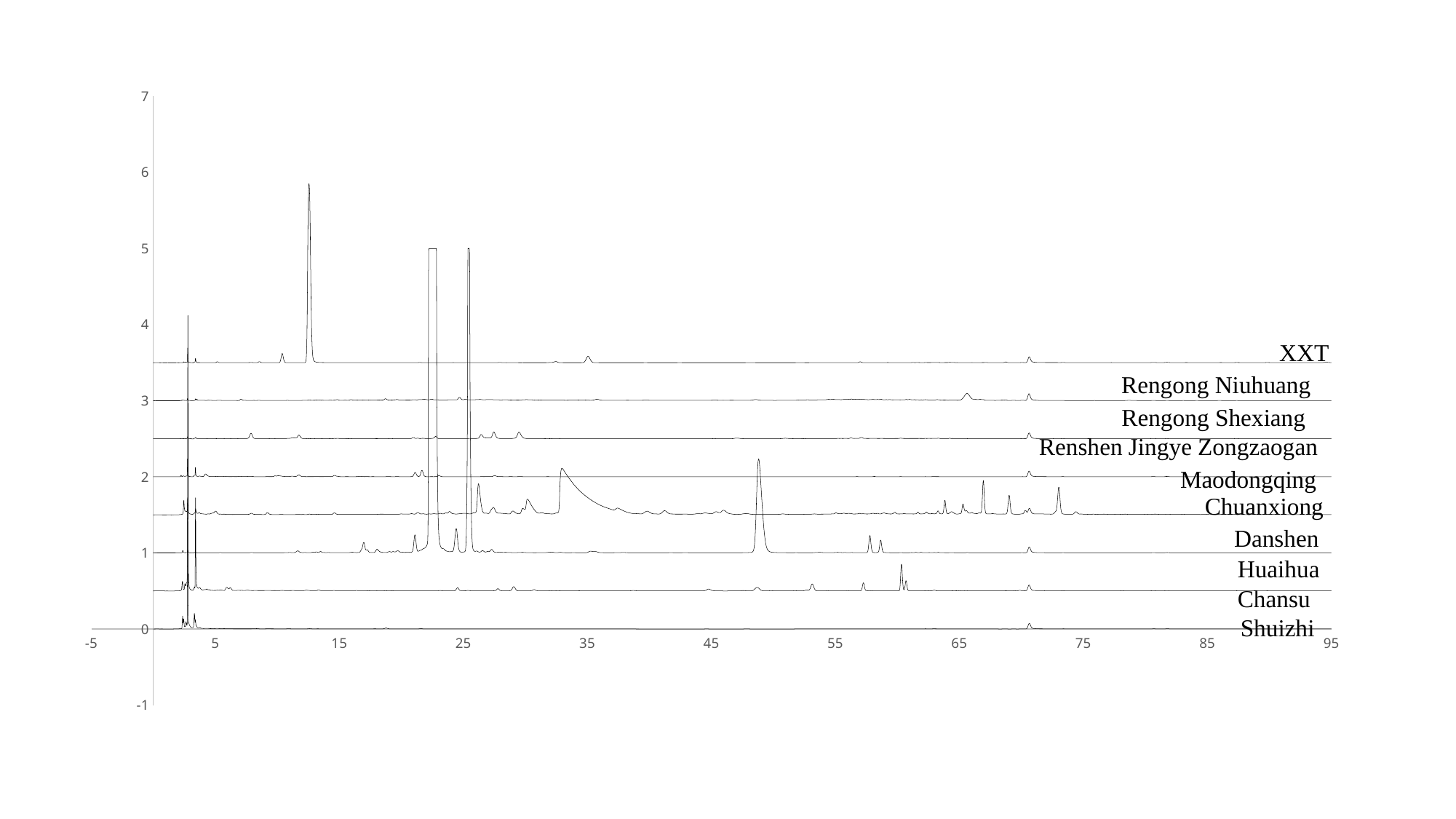

### Chart
| Category | | niuhuang | shexiang | renshen | maodongqing | chuanxiong | danshen | huaihua | chansu | shuizhi |
|---|---|---|---|---|---|---|---|---|---|---|XXT
Rengong Niuhuang
Rengong Shexiang
Renshen Jingye Zongzaogan
Maodongqing
Chuanxiong
Danshen
Huaihua
Chansu
Shuizhi
