# Supplementary material for: Chemical Profiling of Xueshuan Xinmaining Tablet by HPLC and UPLC-ESI-Q-TOF/MS
Source: Evid Based Complement Alternat Med. 2018 Oct 21;2018:2781597. doi: 10.1155/2018/2781597 (PMC6215575; doi:10.1155/2018/2781597)
Supplement: Supplementary Materials — The pharmaceutical manufacture process of XXT described in current Chinese Pharmacopoeia is shown in Figure S1. HPLC of XXT sample and extract of each raw material at 251 nm are shown Figure S2. Relative retention time ratio and relative area ratio of common characteristic peaks in precision, repeatability, and stability test for the HPLC method validation are shown in Tables S1~S6. [file 2781597.f1.zip › 2781597.f1/Supplementary data_ECAM_2499606.docx]

Supplementary data

Chemical Profiling of Xueshuan Xinmaining Tablet by HPLC and UPLC-ESI-Q-TOF/MS

HAN Ya-Xin^1^, WANG Peng-Fei^2,3^, ZHAO Meng^2,4^, CHEN Liang-Mian^2^, WANG Zhi-Min^2^, LIU Xiao-Qian^2^, GAO Hui-Min^2^*, GONG Mu-Xin^1^*, LI Hui^2^, ZHU Ji-Zhong^5^, LIU Chuan-Gui^5^

*^1^ School of Traditional Chinese Medicine*, *Capital Medical University, Beijing 100069, China*

^2^ *Institute of Chinese Materia Medica, China Academy of Chinese Medical Sciences, National Engineering Laboratory for Quality Control Technology of Chinese Herbal Medicine, Beijing 100700, China*

*^3^ Tianjin University of Traditional Chinese Medicine, Tianjin 300193, China*

*^4^ College of Pharmacy, Henan University of Traditional Chinese Medicine, Zhengzhou 450008, China*

*^5^ Jilin Huakang Stock Ltd., Company of Medicines, Dunhua 133700, China*

Corresponding author: Tel/Fax: 86-10-84014128, E-mail: hmgao@icmm.ac.cn (GAO Hui-Min); Tel: 86-10-83911624, [gongmuxin@126.com](mailto:gongmuxin@126.com) (GONG Mu-Xin).


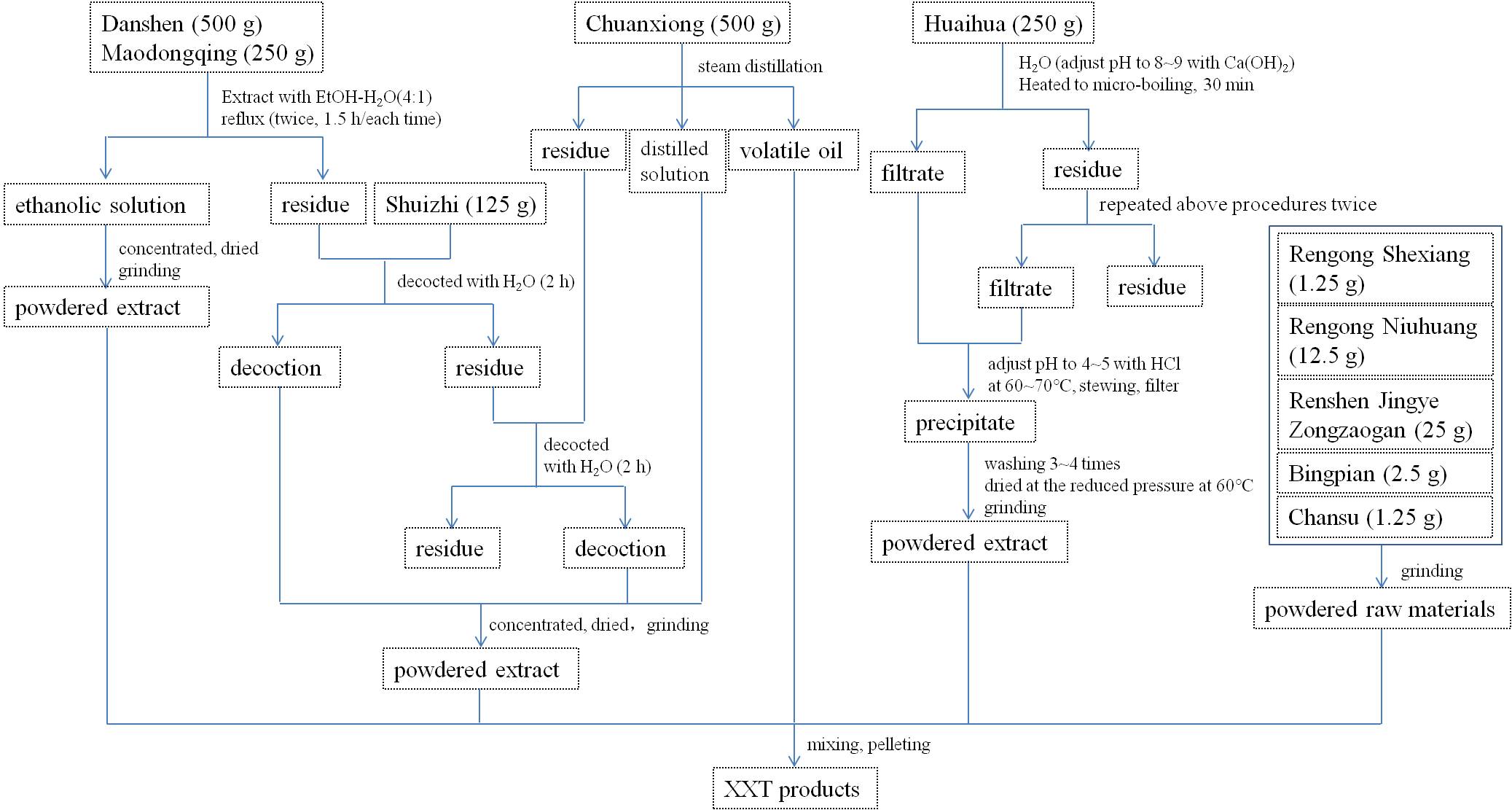


**Figure S1** The pharmaceutical manufacture process of XXT described in current Chinese Pharmacopoeia


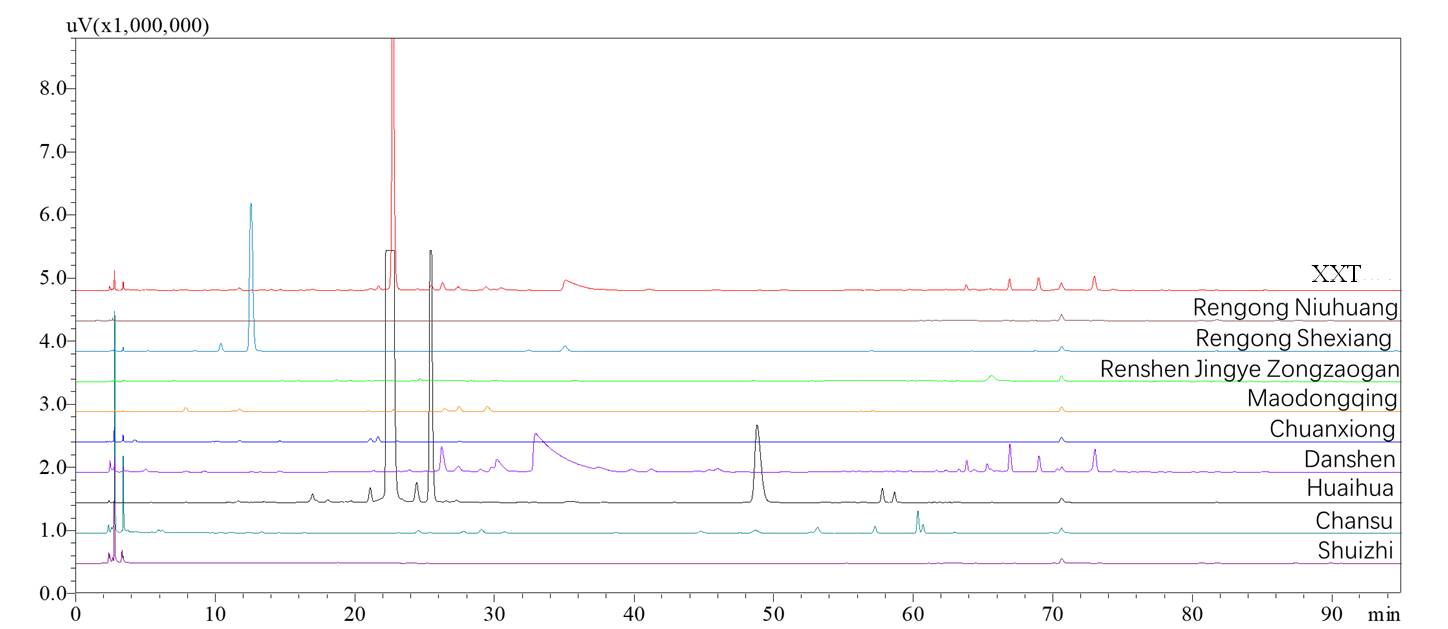


**Figure S2** HPLC of XXT sample and extract of each raw materials at 251 nm.

Table S1 Relative retention time ratio of common characteristic peaks in precision test

|  | **1** | **2** | **3** | **4** | **5** | **6** | **Mean** | **RSD/%** |
| --- | --- | --- | --- | --- | --- | --- | --- | --- |
| **1** | 0.511 | 0.511 | 0.511 | 0.511 | 0.512 | 0.511 | 0.511 | 0.080 |
| **2** | 0.926 | 0.927 | 0.927 | 0.927 | 0.927 | 0.927 | 0.927 | 0.044 |
| **3** | 0.952 | 0.952 | 0.952 | 0.952 | 0.952 | 0.952 | 0.952 | 0.0 |
| **4** | 1.00 | 1.00 | 1.00 | 1.00 | 1.00 | 1.00 | 1.00 | 0.0 |
| **5** | 1.07 | 1.07 | 1.07 | 1.07 | 1.07 | 1.07 | 1.07 | 0.0 |
| **6** | 1.12 | 1.12 | 1.12 | 1.12 | 1.12 | 1.12 | 1.12 | 0.0 |
| **7** | 1.15 | 1.15 | 1.15 | 1.15 | 1.15 | 1.15 | 1.15 | 0.0 |
| **8** | 1.20 | 1.20 | 1.20 | 1.20 | 1.20 | 1.20 | 1.20 | 0.0 |
| **9** | 1.29 | 1.29 | 1.29 | 1.29 | 1.29 | 1.29 | 1.29 | 0.0 |
| **10** | 1.33 | 1.33 | 1.33 | 1.33 | 1.33 | 1.33 | 1.33 | 0.0 |
| **11** | 1.66 | 1.65 | 1.65 | 1.65 | 1.65 | 1.65 | 1.65 | 0.25 |
| **12** | 2.01 | 2.01 | 2.01 | 2.01 | 2.01 | 2.01 | 2.01 | 0.0 |
| **13** | 2.23 | 2.23 | 2.23 | 2.23 | 2.23 | 2.23 | 2.23 | 0.0 |
| **14** | 2.80 | 2.80 | 2.80 | 2.80 | 2.80 | 2.80 | 2.80 | 0.0 |
| **15** | 2.94 | 2.94 | 2.94 | 2.94 | 2.94 | 2.94 | 2.94 | 0.0 |
| **16** | 3.03 | 3.03 | 3.03 | 3.03 | 3.03 | 3.03 | 3.03 | 0.0 |
| **17** | 3.16 | 3.16 | 3.16 | 3.16 | 3.16 | 3.17 | 3.16 | 0.13 |

Table S2 Relative peak area ratio of common characteristic peaks in precision test

|  | **1** | **2** | **3** | **4** | **5** | **6** | **Mean** | **RSD/%** |
| --- | --- | --- | --- | --- | --- | --- | --- | --- |
| **1** | 0.0172 | 0.0172 | 0.0170 | 0.0172 | 0.0173 | 0.0173 | 0.0172 | 0.64 |
| **2** | 0.0178 | 0.0178 | 0.0176 | 0.0177 | 0.0178 | 0.0178 | 0.0178 | 0.47 |
| **3** | 0.0228 | 0.0227 | 0.0223 | 0.0226 | 0.0229 | 0.0228 | 0.0227 | 0.94 |
| **4** | 1.00 | 1.00 | 1.00 | 1.00 | 1.00 | 1.00 | 1.00 | 0.0 |
| **5** | 0.00584 | 0.00584 | 0.00567 | 0.00586 | 0.00584 | 0.00586 | 0.00582 | 1.3 |
| **6** | 0.0229 | 0.0229 | 0.0222 | 0.0229 | 0.0229 | 0.0229 | 0.0228 | 1.3 |
| **7** | 0.0380 | 0.0380 | 0.0370 | 0.0381 | 0.0382 | 0.0382 | 0.0379 | 1.2 |
| **8** | 0.0217 | 0.0215 | 0.0210 | 0.0216 | 0.0216 | 0.0217 | 0.0215 | 1.2 |
| **9** | 0.0230 | 0.0231 | 0.0226 | 0.0233 | 0.0234 | 0.0234 | 0.0231 | 1.3 |
| **10** | 0.0240 | 0.0239 | 0.0235 | 0.0242 | 0.0243 | 0.0244 | 0.0240 | 1.4 |
| **11** | 0.213 | 0.235 | 0.217 | 0.233 | 0.216 | 0.219 | 0.222 | 4.2 |
| **12** | 0.00836 | 0.00837 | 0.00813 | 0.00835 | 0.00836 | 0.00834 | 0.00832 | 1.1 |
| **13** | 0.00873 | 0.00877 | 0.00869 | 0.00810 | 0.00879 | 0.00826 | 0.00856 | 3.5 |
| **14** | 0.0166 | 0.0171 | 0.0171 | 0.0167 | 0.0175 | 0.0167 | 0.0170 | 2.0 |
| **15** | 0.0374 | 0.0385 | 0.0389 | 0.0395 | 0.0396 | 0.0399 | 0.0390 | 2.4 |
| **16** | 0.0480 | 0.0480 | 0.0474 | 0.0468 | 0.0479 | 0.0469 | 0.0475 | 1.2 |
| **17** | 0.0635 | 0.0636 | 0.0633 | 0.0630 | 0.0641 | 0.0635 | 0.0635 | 0.57 |

Table S3 Relative retention time ratio of common characteristic peaks in repeatability test

|  | **1** | **2** | **3** | **4** | **5** | **6** | **Mean** | **RSD/%** |
| --- | --- | --- | --- | --- | --- | --- | --- | --- |
| **1** | 0.514 | 0.514 | 0.513 | 0.513 | 0.514 | 0.514 | 0.514 | 0.10 |
| **2** | 0.930 | 0.928 | 0.928 | 0.928 | 0.928 | 0.929 | 0.928 | 0.090 |
| **3** | 0.955 | 0.953 | 0.954 | 0.953 | 0.953 | 0.954 | 0.954 | 0.086 |
| **4** | 1.00 | 1.00 | 1.00 | 1.00 | 1.00 | 1.00 | 1.00 | 0.0 |
| **5** | 1.08 | 1.08 | 1.08 | 1.08 | 1.08 | 1.08 | 1.08 | 0.0 |
| **6** | 1.12 | 1.12 | 1.12 | 1.12 | 1.12 | 1.12 | 1.12 | 0.0 |
| **7** | 1.16 | 1.15 | 1.15 | 1.15 | 1.15 | 1.15 | 1.15 | 0.35 |
| **8** | 1.21 | 1.20 | 1.20 | 1.20 | 1.20 | 1.20 | 1.20 | 0.34 |
| **9** | 1.30 | 1.29 | 1.29 | 1.29 | 1.29 | 1.29 | 1.29 | 0.32 |
| **10** | 1.34 | 1.34 | 1.34 | 1.34 | 1.34 | 1.34 | 1.34 | 0.00 |
| **11** | 1.66 | 1.65 | 1.65 | 1.65 | 1.65 | 1.65 | 1.65 | 0.25 |
| **12** | 2.02 | 2.01 | 2.01 | 2.01 | 2.02 | 2.02 | 2.02 | 0.27 |
| **13** | 2.24 | 2.22 | 2.23 | 2.23 | 2.23 | 2.23 | 2.23 | 0.28 |
| **14** | 2.81 | 2.80 | 2.81 | 2.80 | 2.80 | 2.80 | 2.80 | 0.18 |
| **15** | 2.95 | 2.94 | 2.95 | 2.94 | 2.94 | 2.94 | 2.94 | 0.18 |
| **16** | 3.04 | 3.03 | 3.04 | 3.03 | 3.03 | 3.03 | 3.03 | 0.17 |
| **17** | 3.22 | 3.20 | 3.21 | 3.21 | 3.21 | 3.21 | 3.21 | 0.20 |

Table S4 Relative peak area ratio of common characteristic peaks in repeatability test

|  | **1** | **2** | **3** | **4** | **5** | **6** | **Mean** | **RSD/%** |
| --- | --- | --- | --- | --- | --- | --- | --- | --- |
| **1** | 0.0135 | 0.0149 | 0.0133 | 0.0147 | 0.0138 | 0.0135 | 0.0140 | 4.9 |
| **2** | 0.0155 | 0.0152 | 0.0147 | 0.0157 | 0.0161 | 0.0154 | 0.0154 | 3.1 |
| **3** | 0.0205 | 0.0205 | 0.0205 | 0.0203 | 0.0212 | 0.0205 | 0.0206 | 1.5 |
| **4** | 1.00 | 1.00 | 1.00 | 1.00 | 1.00 | 1.00 | 1.00 | 0.0 |
| **5** | 0.00492 | 0.00547 | 0.00536 | 0.00515 | 0.00534 | 0.00516 | 0.00523 | 3.8 |
| **6** | 0.0204 | 0.0218 | 0.0215 | 0.0205 | 0.0211 | 0.0209 | 0.0210 | 2.6 |
| **7** | 0.0333 | 0.0361 | 0.0354 | 0.0348 | 0.0346 | 0.0344 | 0.0348 | 2.7 |
| **8** | 0.0182 | 0.0203 | 0.0195 | 0.0196 | 0.0192 | 0.0191 | 0.0193 | 3.6 |
| **9** | 0.0204 | 0.0222 | 0.0213 | 0.0214 | 0.0209 | 0.0210 | 0.0212 | 2.8 |
| **10** | 0.0220 | 0.0220 | 0.0205 | 0.0217 | 0.0197 | 0.0202 | 0.0210 | 4.8 |
| **11** | 0.213 | 0.235 | 0.217 | 0.233 | 0.216 | 0.219 | 0.222 | 4.2 |
| **12** | 0.00741 | 0.00720 | 0.00706 | 0.00759 | 0.00764 | 0.00687 | 0.00730 | 4.2 |
| **13** | 0.00702 | 0.00774 | 0.00747 | 0.00742 | 0.00752 | 0.00740 | 0.00743 | 3.2 |
| **14** | 0.0170 | 0.0182 | 0.0172 | 0.0168 | 0.0160 | 0.0174 | 0.0171 | 4.2 |
| **15** | 0.0395 | 0.0420 | 0.0369 | 0.0390 | 0.0390 | 0.0402 | 0.0394 | 4.2 |
| **16** | 0.0440 | 0.0464 | 0.0462 | 0.0439 | 0.0448 | 0.0452 | 0.0451 | 2.4 |
| **17** | 0.0611 | 0.0625 | 0.0615 | 0.0581 | 0.0583 | 0.0604 | 0.0603 | 2.9 |

Table S5 Relative retention time ratio of common characteristic peaks in stability test

|  | **0 h** | **4 h** | **8 h** | **12 h** | **18 h** | **24 h** | **Mean** | **RSD/%** |
| --- | --- | --- | --- | --- | --- | --- | --- | --- |
| **1** | 0.511 | 0.511 | 0.511 | 0.512 | 0.513 | 0.513 | 0.512 | 0.19 |
| **2** | 0.924 | 0.926 | 0.927 | 0.927 | 0.927 | 0.927 | 0.926 | 0.13 |
| **3** | 0.951 | 0.952 | 0.952 | 0.952 | 0.952 | 0.953 | 0.952 | 0.066 |
| **4** | 1.00 | 1.00 | 1.00 | 1.00 | 1.00 | 1.00 | 1.00 | 0.0 |
| **5** | 1.07 | 1.07 | 1.07 | 1.07 | 1.08 | 1.07 | 1.07 | 0.38 |
| **6** | 1.12 | 1.12 | 1.12 | 1.12 | 1.12 | 1.12 | 1.12 | 0.0 |
| **7** | 1.15 | 1.15 | 1.15 | 1.15 | 1.15 | 1.15 | 1.15 | 0.0 |
| **8** | 1.20 | 1.20 | 1.20 | 1.20 | 1.20 | 1.20 | 1.20 | 0.0 |
| **9** | 1.28 | 1.29 | 1.29 | 1.29 | 1.29 | 1.29 | 1.29 | 0.32 |
| **10** | 1.32 | 1.33 | 1.33 | 1.33 | 1.33 | 1.33 | 1.33 | 0.31 |
| **11** | 1.63 | 1.65 | 1.65 | 1.65 | 1.65 | 1.64 | 1.64 | 0.51 |
| **12** | 2.00 | 2.01 | 2.01 | 2.01 | 2.01 | 2.01 | 2.01 | 0.20 |
| **13** | 2.21 | 2.23 | 2.23 | 2.23 | 2.23 | 2.23 | 2.23 | 0.37 |
| **14** | 2.78 | 2.80 | 2.80 | 2.80 | 2.80 | 2.80 | 2.80 | 0.29 |
| **15** | 2.92 | 2.94 | 2.94 | 2.94 | 2.93 | 2.94 | 2.94 | 0.29 |
| **16** | 3.01 | 3.03 | 3.03 | 3.03 | 3.03 | 3.03 | 3.03 | 0.27 |
| **17** | 3.18 | 3.20 | 3.21 | 3.21 | 3.20 | 3.20 | 3.20 | 0.34 |

Table S6 Relative peak area ratio of common characteristic peaks in stability test

|  | **0 h** | **4 h** | **8 h** | **12 h** | **18 h** | **24 h** | **Mean** | **RSD/%** |
| --- | --- | --- | --- | --- | --- | --- | --- | --- |
| **1** | 0.0173 | 0.0172 | 0.0170 | 0.0173 | 0.0176 | 0.0173 | 0.0173 | 1.1 |
| **2** | 0.0164 | 0.0178 | 0.0176 | 0.0178 | 0.0179 | 0.0177 | 0.0175 | 3.2 |
| **3** | 0.0242 | 0.0228 | 0.0223 | 0.0229 | 0.0229 | 0.0226 | 0.0229 | 2.8 |
| **4** | 1.00 | 1.00 | 1.00 | 1.00 | 1.00 | 1.00 | 1.00 | 0.0 |
| **5** | 0.00594 | 0.00584 | 0.005667 | 0.00584 | 0.00601 | 0.00583 | 0.00586 | 2.0 |
| **6** | 0.0229 | 0.0229 | 0.0222 | 0.0229 | 0.0230 | 0.0224 | 0.0227 | 1.5 |
| **7** | 0.0367 | 0.0380 | 0.0370 | 0.0382 | 0.0384 | 0.0376 | 0.0376 | 1.8 |
| **8** | 0.0206 | 0.0217 | 0.0210 | 0.0216 | 0.0218 | 0.0213 | 0.0213 | 2.2 |
| **9** | 0.0219 | 0.0230 | 0.0226 | 0.0234 | 0.0234 | 0.0229 | 0.0229 | 2.5 |
| **10** | 0.0246 | 0.0240 | 0.0235 | 0.0243 | 0.0241 | 0.0235 | 0.0240 | 1.8 |
| **11** | 0.249 | 0.254 | 0.248 | 0.256 | 0.258 | 0.252 | 0.253 | 1.6 |
| **12** | 0.00853 | 0.00836 | 0.00813 | 0.00836 | 0.0086 | 0.00844 | 0.00840 | 2.0 |
| **13** | 0.00891 | 0.00873 | 0.00869 | 0.00879 | 0.0091 | 0.00896 | 0.00886 | 1.8 |
| **14** | 0.01654 | 0.0166 | 0.0171 | 0.0175 | 0.0176 | 0.0172 | 0.0171 | 2.6 |
| **15** | 0.0378 | 0.0385 | 0.0389 | 0.0396 | 0.0398 | 0.0390 | 0.0389 | 1.9 |
| **16** | 0.04720 | 0.0480 | 0.0474 | 0.0479 | 0.0479 | 0.0471 | 0.0476 | 0.83 |
| **17** | 0.0620 | 0.0635 | 0.0633 | 0.0641 | 0.0645 | 0.0634 | 0.0635 | 1.3 |
